# Deep Beats, Deep Thoughts? Predicting General Cognitive Ability from Natural Music-Listening Behavior

**DOI:** 10.3390/jintelligence14020029

**Published:** 2026-02-13

**Authors:** Larissa Sust, Maximilian Bergmann, Markus Bühner, Ramona Schoedel

**Affiliations:** 1 Department of Psychology, Ludwig-Maximilians-Universität München, 80802 Munich, Germany; buehner@psy.lmu.de (M.B.); ramona.schoedel@charlotte-fresenius-uni.de (R.S.); 2 Institute of Behavioral Science and Technology, University of St. Gallen, 9000 St. Gallen, Switzerland; maximilian.bergmann@unisg.ch; 3 Department of Psychology, Charlotte Fresenius Hochschule, University of Psychology, 80797 Munich, Germany

**Keywords:** music listening, general cognitive ability, mobile sensing

## Abstract

Music is more than just entertainment. It is a complex auditory stimulus that engages various cognitive processing systems. Accordingly, natural music-listening patterns may reveal insights into individual differences in general cognitive ability (GCA). In this study (*N* = 185), we used real-world smartphone-based music-listening records collected over five months to explore this question. We quantified participants’ listening habits (e.g., listening durations) and music preferences based on audio characteristics (e.g., tempo, mode) and lyrical characteristics (e.g., positive emotion words, affiliation words) of the songs they had listened to. These strictly behavioral features were used to predict GCA scores using linear LASSO regression and nonlinear random forest models. Out-of-sample cross-validation indicated modest predictive performance, with only the random forest model detecting small but reliable associations between music-listening behavior and GCA. Interpretable machine learning analyses showed that lyrics-based preferences were the most informative feature group, followed by listening habits, whereas audio characteristics contributed little predictive value. We discuss how these findings offer initial evidence that cognitive ability may be reflected, albeit subtly, in micro-patterns of everyday, non-achievement-related behavior, and outline conceptual and methodological challenges for future work using digital behavioral data to complement traditional cognitive assessment.

## 1. Introduction

The Cattell–Horn–Carroll theory is the most widely accepted and comprehensive framework for describing the structure of human cognitive abilities (McGrew, 2005; Schneider & McGrew, 2018). It organizes numerous narrow cognitive processes into several broad abilities, such as fluid and crystallized intelligence, which converge onto a higher-order general factor (Jensen, 1998). This general cognitive ability (GCA) represents “a distillate of the common source of individual differences in all mental tests, completely stripped of their distinctive features of information content, skill, strategy, and the like” (Jensen, 1998, p. 74). GCA reflects the capacity to think rationally, act purposefully, and adapt effectively to the cognitive complexity of everyday life (Gottfredson, 1997; Stasielowicz, 2020; Wechsler, 1944) and robustly predicts educational attainment (Deary et al., 2007; Lozano-Blasco et al., 2022; Roth et al., 2015), occupational success (Ackerman & Heggestad, 1997; Hunter & Schmidt, 1996; Schmidt & Hunter, 2004), and physical and mental health outcomes (Deary et al., 2021; Fries et al., 2025).

Traditionally, GCA is assessed using formal performance tests administered in controlled laboratory or achievement-oriented settings (Flanagan & McDonough, 2018; Koch et al., 2021). Yet, cognitive abilities are also theorized to manifest in everyday behavior (Gottfredson, 1997; Schultheis & Cooper, 2022). Prior research supports this view by linking functional performance—such as preparing food, navigating maps, or understanding news articles—to intelligence (Allaire & Marsiske, 1999; Gottfredson, 1997). With the increasing digitalization of daily life, performance in digital environments, such as laptop interaction (Vuong et al., 2024) and smartphone use (Gordon et al., 2019; Rauber et al., 2019), has also been examined as an indicator of cognitive functioning or decline. However, cognitive abilities have rarely been examined in non-achievement-related, everyday contexts. Only a handful of studies, primarily from computational sciences, have attempted to predict cognitive abilities from naturally occurring digital data sources such as social media activity (Kosinski et al., 2013; Settanni et al., 2018; Wei & Stillwell, 2017). This gap highlights the need to better understand how intelligence is reflected in routine behaviors that are not directly tied to performance or evaluation. 

As everyday behaviors increasingly leave digital traces, it has become more feasible to examine how psychological differences manifest in daily life (Boyd et al., 2020; Renner et al., 2020), introducing a level of ecological assessment that was largely inaccessible in earlier decades (Baumeister et al., 2007; Furr, 2009). In this context, combining digital behavioral data with state-of-the-art machine learning offers a powerful approach for uncovering subtle and potentially complex behavioral indicators of individual differences, as repeatedly proposed in personality research (e.g., Kosinski et al., 2016; Peters et al., 2022; Phan & Rauthmann, 2021). Extending this logic to cognitive abilities, demonstrating that GCA can be inferred from everyday digital behavior would not only enable low-threshold, ecologically valid proxies for cognitive assessment—supporting, for example, adaptive digital environments or early intervention strategies (Koch et al., 2021)—but would also deepen our theoretical understanding of what intelligence is and how it manifests in real-world settings.

In the present work, we focus on music-listening behavior as a promising candidate behavior for predicting GCA. Music listening is one of the most ubiquitous leisure activities in modern life (IFPI, 2023) and constitutes a cognitively and emotionally stimulating experience (Juslin & Laukka, 2004; North & Hargreaves, 1999). Neuroscientific research shows that listening to music engages widespread brain networks involved in auditory processing, emotion, working memory, and reward (e.g., Blood & Zatorre, 2001; Janata et al., 2002; Koelsch et al., 2005; Menon & Levitin, 2005). Complementing this, cognitive studies have demonstrated that music can influence performance on various cognitive tasks (Furnham & Bradley, 1997; Salamé & Baddeley, 1989; Schellenberg et al., 2007). Together, these lines of research indicate that musical experiences are intertwined with cognitive functioning. More broadly, the personality trait Openness—often theoretically and empirically linked to intelligence (e.g., Anglim et al., 2022; DeYoung, 2015)—is also a correlate of musical preferences (I. Anderson et al., 2020; Greenberg et al., 2016; Rentfrow & Gosling, 2003; Sust et al., 2023).

Yet only a few studies have directly examined cognitive abilities in relation to music listening, finding that music preferences differed meaningfully by ability level (Bonetti & Costa, 2016; George et al., 2007; Kanazawa & Perina, 2012; Pirgon, 2021; Račevska & Tadinac, 2019; Rentfrow & Gosling, 2003). For example, Rentfrow and Gosling (2003) showed that people with higher analytic reasoning ability preferred more reflective and complex (e.g., Jazz) as well as intense and rebellious musical genres (e.g., Rock). However, these studies typically relied on self-reports or laboratory experiments, which may not accurately reflect natural listening behavior due to recall errors, social desirability bias, or artificial conditions (Baumeister et al., 2007; Greenberg & Rentfrow, 2017; Paulhus & Vazire, 2007). Consequently, it remains unclear how cognitive abilities relate to actual music-listening behavior in everyday life.

Theoretical perspectives such as the Uses and Gratifications framework propose that individuals actively select music to satisfy specific psychological needs (DeNora, 2000; Katz et al., 1973). In line with this, more intelligent individuals tend to engage with music for cognitively oriented purposes (i.e., as an intellectual experience) rather than primarily for emotional arousal or background entertainment (Bonetti et al., 2021; Chamorro-Premuzic & Furnham, 2007). Thus, intelligence may be expressed not only in the genres people prefer but also in the melodic and lyrical characteristics of the songs they choose in real life.

Importantly, non-instrumental songs consist of both melodic and lyrical components, which are processed independently in the brain and contribute uniquely to the listening experience (Ali & Peynircioğlu, 2006; C. A. Anderson et al., 2003; Besson et al., 1998; Bonnel et al., 2001). Recent work shows that audio and lyric features relate differentially to personality traits (Sust et al., 2023), suggesting that both may provide complementary information about GCA, too.

Because digitalization has transformed how people listen to music, most listening now occurs on digital devices that automatically record when and what individuals play (I. Anderson et al., 2020; Greenberg & Rentfrow, 2017). Smartphones, the primary devices for music consumption (IFPI, 2023), are sensor-rich tools uniquely suited to unobtrusively capturing music-listening data in real-world settings (Sust et al., 2023). Meanwhile, advances in machine learning have enabled the efficient, large-scale analysis of such rich behavioral data (Kosinski et al., 2016; Peters et al., 2022). Music preferences can now be quantified through the intrinsic melodic and lyrical properties of the songs people play, derived via computational music information retrieval and natural language processing, rather than relying on subjective ratings or inconsistent genre labels (Aucouturier & Pachet, 2003; Fricke et al., 2018; Sust et al., 2023). These developments enable out-of-sample prediction approaches, offering a powerful way to estimate the accuracy with which natural listening patterns predict cognitive abilities.

In the present study, we examined whether GCA can be predicted from natural music-listening behavior on smartphones in a sample of 185 participants. Using ecologically valid music-listening records collected over five months, we enriched each played song with audio and lyric characteristics, yielding 215 features that capture participants’ music preferences and listening habits. These features served as predictors in linear LASSO regression and non-linear random forest models. We evaluated out-of-sample predictive performance and compared the independent contributions of audio- and lyrics-based preferences. By linking digital music-listening behavior to cognitive ability, this study extends research on music and intelligence beyond self-reports and experimental tasks, offering an ecologically valid, data-driven approach to understanding cognitive differences in everyday life and providing a starting point for using digital behavioral data for inferring cognitive abilities.

## 2. Materials and Methods

We reused data collected in our Smartphone Sensing Panel Study, an interdisciplinary research project conducted by LMU Munich in cooperation with the Leibniz Institute for Psychology (Schoedel & Oldemeier, 2020). The study collected a multi-method dataset by integrating online surveys, experience sampling, and mobile sensing. All procedures were approved by the Ethics Committee of LMU Munich under the study title “A longitudinal panel study combining smartphone sensing and survey methods” and were conducted in accordance with the Declaration of Helsinki and the General Data Protection Regulation (GDPR). Written informed consent was obtained from all participants involved in the study. In the present manuscript, we describe only the procedures and measures from the study that are relevant to the current research question. Further details on all study procedures are provided in the preregistered study protocol (Schoedel & Oldemeier, 2020) and related publications (Große Deters & Schoedel, 2024; Reiter & Schoedel, 2024; Schoedel et al., 2023). While we cannot share raw sensing data for privacy reasons, aggregated data and all analysis scripts used in this project are openly available, along with our online Supplementary Materials, in our repository, under https://osf.io/cz4dj/ (accessed on 6 February 2026).

### 2.1. Procedure

The initial dataset comprised 850 participants, sampled using quotas to represent the German population with respect to gender, age, education, income, religion, and relationship status. Participants were—by requirement—between 18 and 65 years old, fluent in German, and the sole user of a smartphone running on Android version 5 or higher (Schoedel & Oldemeier, 2020). Data collection started in May 2020 and lasted for either three (*n* = 191) or six months (*n* = 659), depending on a random group assignment. All data were collected using the PhoneStudy research application (short: app), which participants installed on their private smartphones at study onset. Subsequently, the app began logging mobile-sensing data continuously, including music-listening records, for the duration of the study. In addition, depending on the group assignment, the app administered three or six monthly online surveys, each measuring different psychological constructs, including general cognitive ability (GCA), and one or two 14-day experience-sampling waves. For a comprehensive overview of all collected measures and the timeline, please refer to the study protocol by Schoedel and Oldemeier (2020).

### 2.2. Participants

In the present study, we used questionnaire data collected during Survey 1 (May 2020, demographics) and Survey 5 (September 2020, GCA), as well as the sensing data collected prior to Survey 5 (May–September 2020). After applying several exclusion criteria, the resulting sample comprised *N* = 185. First, we excluded participants who did not complete the intelligence test (*n* = 467), either due to the random group assignment (participants in the three-month group never received Survey 5) or due to non-compliance. Next, we excluded test takers (*n* = 3) who showed clear signs of careless responding, defined as a pattern of repeatedly selecting the same response option paired with extremely short response times on at least three items in any given subtest. Extremely short response times were defined relative to the sample distribution as less than one-third of the median response time of the fastest item in the respective subtest. Finally, we excluded participants without any recorded music-listening events, that is, those who had not listened to music on their smartphones (*n* = 195). In the final sample, 163 participants provided demographic information. Participants’ ages ranged from 18 to 64 (*M* = 37.2, *SD* = 12.7), and 46% (*n* = 75) identified as women, 54% (*n* = 88) as men, and no one identified as diverse. Demographic variables were used for descriptive purposes only in the present study. Regarding educational attainment, 1% had no school-leaving certificate, 9% had a lower secondary school certificate (“Hauptschulabschluss”), 31% had an intermediate secondary school certificate (“Realschulabschluss”), 35% had the highest secondary school certificate (“Abitur”), 23% had a university degree, and 1% had completed a doctorate.

### 2.3. Measures

#### 2.3.1. General Cognitive Ability

We assessed GCA using a short version of the Inventory for Testing Cognitive Abilities (INT), developed explicitly for smartphone-based administration (Schuhfried, 2022). In line with the Cattell–Horn–Carroll model of cognitive abilities (Schneider & McGrew, 2018), the INT measures indicators of three broad abilities: fluid reasoning, comprehension knowledge, and quantitative knowledge (Schuhfried, 2022). In the current full-length version of the test, which was developed after our data collection, visual processing is included as a fourth ability.

Fluid reasoning was assessed via a matrices task, comprehension knowledge via an analogies task, and quantitative knowledge via a task requiring the insertion of arithmetic symbols into equations. These conventional intelligence test formats were adapted for mobile delivery by standardizing item layout and size across devices (Schuhfried, 2022). For example, in the matrices task, participants indicated which of nine matrices did not fit, rather than selecting a missing matrix. The adapted version was validated across multiple devices, both with and without supervision, and showed no systematic performance differences. The short version used in the present study comprised 20 items and was administered linearly with no time limit. According to the manual, the INT follows the linear logistic model of item response theory (Rasch, 1960) and shows no systematic performance differences by age, gender, education, or device (Schuhfried, 2022). In our sample, none of the three subtests nor the combined test showed significant deviations from the Rasch model, as assessed by a bootstrapped goodness-of-fit test in the R package ltm (version 1.2-0; Rizopoulos, 2006). However, reliability estimates for the subtests were modest, with Cronbach’s *α* = 0.56 [0.49, 0.63] for fluid reasoning, 0.70 [0.65, 0.75] for comprehension knowledge, and 0.64 [0.58, 0.70] for quantitative knowledge. Only the combined test demonstrated satisfactory internal consistency, *α* = 0.80 [0.77, 0.83]. We therefore used the combined GCA score and did not analyze the subtest scores separately. To avoid data leakage in our subsequent resampling procedure, we did not compute Rasch-based person parameters. Instead, we used the sum of correctly solved items as the measure of intelligence.

#### 2.3.2. Music-Listening Measures

The PhoneStudy app passively recorded various aspects of participants’ smartphone usage, including their music-listening behavior. The app generated a time-stamped event log for each played song, specifying the song’s title, artist, and album. To derive meaningful music preference variables from these raw event logs, we applied a preprocessing pipeline similar to that used by Sust et al. (2023). As an initial step, we identified and labelled non-musical tracks (e.g., audiobooks), which made up 32% of all events in the logs, leaving a total of 58.247 unique songs played in our sample. We then enriched these songs with additional information from the music-streaming service Spotify and the crowdsourced music database Genius through API calls. From Spotify, we obtained 11 features, representing technical audio characteristics (e.g., “tempo,” “acousticness”) derived from the songs’ audio recordings (Spotify, 2025). From Genius, we retrieved the songs’ lyrics. To extract psychologically meaningful characteristics from the lyrics, we applied the Linguistic Inquiry and Word Count (LIWC; Meier et al., 2019; Tausczik & Pennebaker, 2010). LIWC analyzes text by comparing each word to a validated dictionary of psychologically relevant categories and calculating the relative frequency of words within each category. These categories represent linguistic dimensions (e.g., verbs, first-person singular pronouns) as well as psychological constructs such as anger (e.g., hate, mad), power (e.g., own, order), friends (e.g., friend, boyfriend), leisure (e.g., game, party), and death (e.g., die, kill). After removing 12 punctuation-related categories and one emoji category—excluded because punctuation and emoji use are distorted in song lyrics—LIWC provided 81 features in total. We applied language-specific LIWC versions separately to English (75%) and German (19%) lyrics (Meier et al., 2019; Tausczik & Pennebaker, 2010), which accounted for the majority of songs in the corpus and are assumed to be understood by our German-speaking sample. In addition, we adopted one feature indicating the language of each song’s lyrics. In total, this preprocessing pipeline yielded 93 features quantifying various musical characteristics, which we assigned to the respective songs in the raw event logs. However, not all music-listening events could be enriched in that manner because some lacked clear song information (e.g., title and artist in a single field) or were not covered by the corresponding online sources. Out of the 58.247 unique songs in our sample, 87% were correctly matched with audio features and 45% with lyric features. All matches were validated both technically and manually.

In the final preprocessing step, we aggregated these song-level features at the person level. For each participant, we filtered for musical tracks that lasted longer than 20 s, excluding non-musical tracks and skipped songs. We then aggregated the distribution of each song-level feature over all played songs via the median and median absolute deviation (for numeric variables) or percentage scores (for factor variables). The resulting features covered the average and variance in music preferences for (1) audio characteristics (e.g., mean tempo of played songs; n = 31) and (2) lyrics characteristics (e.g., variance in family-related lyrics; n = 162). As the external song-level characteristics were not available for all songs, the music preference features only covered a portion of participants’ played tracks (*M* = 84.7%, *SD* = 19.3 for Spotify-based features; *M* = 59.7% and *SD* = 23.0 for lyrics-based features). To account for the limited coverage, we created one additional feature for each preference group, indicating the proportion of participants’ songs covered. In addition, we extracted 20 listening-habit features that quantified the extent of participants’ music consumption (e.g., the total number of played songs). In total, we obtained 215 features capturing participants’ music-listening behaviors, which served as predictors in our intelligence predictions.

### 2.4. Analytic Strategy

#### 2.4.1. Machine Learning

Based on the music-listening features, we trained machine learning models to predict the GCA score. All machine learning analyses were conducted within the mlr3verse (version 0.2.8, Lang & Schratz, 2022).

We performed target-independent preprocessing of the music-listening features using the caret package (version 6.0-94, Kuhn, 2008). We removed extreme outliers (mean ± 4 standard deviations) and excluded all features with high missingness (90% cut-off), near-/zero variance (10% cut-off), and high intercorrelations (*r* > 0.90; Kuhn & Johnson, 2013), resulting in a final set of 197 predictor variables. Furthermore, we performed scaling and histogram imputation of missing values, which we integrated into the resampling scheme to avoid overfitting.

We trained regularized linear regression models (LASSO; Tibshirani, 1996) and non-linear tree-based random forest models (Breiman, 2001). We selected these two algorithms for their model-inherent feature selection, which enables them to handle high-dimensional, intercorrelated predictor spaces, and for their effectiveness in prior psychological research (Pargent et al., 2023). LASSO models were trained using the optimal regularization parameter selected via internal cross-validation (*λ_min_*) as implemented in glmnet (version 4.1-8, Friedman et al., 2010). Random forest models were trained with default hyperparameter settings using ranger (version 0.16.0, Wright & Ziegler, 2017). For benchmarking, we also trained a featureless baseline model that predicted the mean of the training data’s GCA scores for all observations in the respective test set, without considering any features.

We conducted a benchmark experiment to compare the predictive performance of the LASSO and random forest models against the baseline. To estimate out-of-sample predictive performance, we split the data into training and test sets using 20-times repeated 5-fold cross-validation (20 × 5 CV). We chose this strategy to ensure reasonably large test sets, given our total sample size (185/5 = 37). We evaluated model performances by averaging the Spearman rank order correlation coefficients (*ρ*) obtained in each of the 20 × 5 CV iterations. These coefficients indicate the association between the actual and predicted intelligence scores in each iteration, whereby higher correlations indicate better predictions. Models with a median *ρ* above zero were considered predictive as they learned signal in the features. Additionally, we report the average mean squared error (MSE) as a secondary performance measure. We did not conduct significance tests of model performance as machine learning approaches are primarily intended to estimate predictive performance and generalization (Pargent et al., 2023), and because the present study was exploratory and not preregistered.

#### 2.4.2. Model Interpretations

To gain further insights into the best prediction model, we conducted an interpretable machine learning analysis to compare the unique contributions of the different feature groups (i.e., audio preferences, lyrics preferences, and listening habits) and individual features. We used out-of-sample permutation-based feature importances to estimate how much information the models learned from each feature (group) when trying to predict unseen GCA scores. We based these analyses on the random forest model, which outperformed the LASSO as reported in the results section below.

To understand the unique relevance of the different groups of music-listening features, we obtained the permutation-based loss in *ρ* for each feature group (i.e., drop loss). For each model in our 20 × 5 CV benchmark, we conducted 10 permutations for each feature group, computed the corresponding drop-loss performances, and aggregated them to the median loss per model. To obtain robust estimates of average grouped feature importance, we then aggregated the median drop-loss by feature group across all 20 × 5 models. This metric represents the expected decline in prediction performance when permuting the features of the respective group, with stronger negative values indicating a higher importance of the group for the prediction. For calculating individual feature importances, we applied the same procedure and permuted every feature 10 times in each of the 20 × 5 models. The median drop-loss across all models represents the unique importance of each feature for the prediction performance.

### 2.5. Statistical Software

Our analyses were conducted in the statistical software R (version 4.5.1 for preprocessing, R Core Team, 2025; version 4.0.4. for machine learning analysis, R Core Team, 2021). We used self-written functions (see Schoedel et al., 2025) and the package dplyr (version 1.0.7, Wickham et al., 2021) for extracting person-level variables. For predictive modeling, we employed the packages mlr3 (version 0.16.1, Lang et al., 2019), glmnet (version 4.1-8, Friedman et al., 2010), and ranger (version 0.16.0, Wright & Ziegler, 2017). Furthermore, we used iml (version 0.11.1, Molnar et al., 2018) for interpretable machine learning.

## 3. Results

### 3.1. Descriptive Statistics

Across the five-month study period, participants listened to music on an average of 32% of study days (*SD* = 0.3) and played an average of 696 songs (*SD* = 1199). The most frequently used music applications were Spotify (44%), followed by Google Play Music (14%) and Amazon Music (12%). Participants exhibited a mean performance of *M* = 10.64 (*SD* = 4.13) on the general cognitive ability (GCA) measure, with a score of 20 representing the maximum performance. GCA correlated negatively with age (*r*(161) = −0.33, 95% CI [−0.46; −0.18], *p* < .000) and positively with education (*r*(161) = 0.41, 95% CI [0.27; 0.53], *p* < .000), but did not differ by gender (*M_m_* = 10.78, *M_f_* = 11.17, *t*(157) = −0.65, *p* = 0.52). Summary statistics for all music-listening features and their zero-order correlations with GCA and the demographic variables are presented in the online Supplementary Materials (Tables S1 and S2).

### 3.2. Predictions of General Cognitive Ability

**Figure 1:**
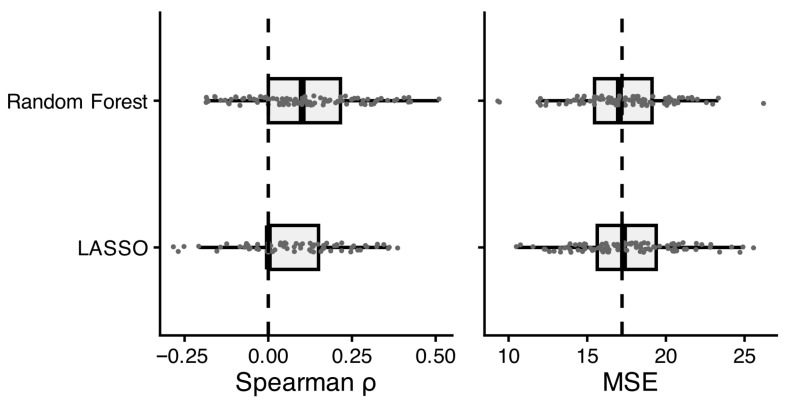
Distribution of prediction performance for general cognitive ability across the 100 iterations of repeated cross-validation (20 × 5) by models. The middle symbol represents the median, and boxes include values between the 25% and 75% quantiles. The dotted line indicates the performance of the featureless baseline model.

We examined whether general cognitive ability could be predicted from behavioral music-listening features obtained via mobile sensing. Out-of-sample prediction performance for all models is shown in Figure 1 and summarized in Table A1 in Appendix A. Overall, prediction accuracy was low. The linear LASSO models failed to produce meaningful predictions (*ρ_md_* = 0.00), with strong regularization shrinking the features’ coefficients toward zero in cross-validation, likely due to weak signal and limited sample sizes per training fold. In contrast, the nonlinear random forest models yielded a small positive median correlation between predicted and observed GCA (*ρ_md_* = 0.10), indicating that they were better able to aggregate weak, distributed, and potentially nonlinear signals across features. Nevertheless, random forest predictions were not uniformly successful: some cross-validation iterations produced negative correlations, and the model’s mean squared error (MSE) closely approximated that of the featureless baseline model. This pattern suggests that, on average, predicting GCA using music-listening features was only slightly more informative than using the sample mean. Given that only the random forest models showed predictive value, subsequent interpretations focus exclusively on these models.

### 3.3. Interpretation of Prediction Models

To understand which aspects of music listening contributed most strongly to predicting GCA, we examined both the importance of feature groups and the importance of individual features.

**Figure 2:**
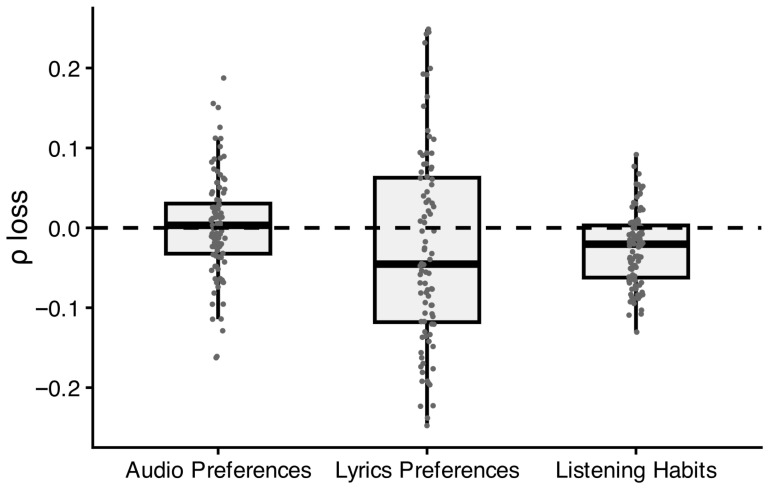
Distribution of permutation-based grouped feature importance in terms of *ρ*-loss across the 100 resampling iterations in random forest models. Importance scores for each iteration are based on median *ρ*-loss across 10 random permutations. The middle symbol represents the median, and boxes include values between the 25% and 75% quantiles. The dotted line indicates an importance of zero, that is, no loss in *ρ* when perturbing the features of the respective group.

Permutation-based feature grouped importance scores (Figure 2; Table A2) revealed that lyrics-based preferences were the most influential predictors of GCA in the random forest models, followed by listening habits. In contrast, audio-based preferences contributed the least, as excluding audio features did not meaningfully reduce predictive performance. These findings suggest that the linguistic properties of listened-to songs were more strongly associated with GCA than their melodic properties.

Additionally, we identified the ten most informative individual features for predicting GCA (Table 1), with the full feature-importance rankings reported in Table S3 of our online Supplementary Materials. Only one audio feature appeared among the top predictors, but it was the most influential one overall: average liveness, reflecting the probability that a song’s recording contains an audience, was most relevant for random forest models. Correlation coefficients indicate that higher liveness values were associated with lower predicted GCA scores. Seven of the remaining top features came from the lyrics preferences, consistent with the grouped importance results. Lyrics containing more social words (e.g., help, friend) or greater variability in social words, more tentative language (e.g., if, any, or), and more positive emotional tone tended to predict lower GCA scores. In contrast, lyrics with stronger present-focus (e.g., are, can), greater authenticity (i.e., perceived honesty), and more home-related words (e.g., house, bed) predicted higher GCA scores (for an in-depth explanation of the Linguistic Inquiry and Word Count categories, see Tausczik & Pennebaker, 2010). Finally, two listening-habit features were among the top predictors: a lower percentage of German-language songs and longer overall listening durations were associated with higher predicted GCA scores.

## 4. Discussion

In the present study, we employed a machine learning approach to examine how well general cognitive ability (GCA) can be predicted from everyday behavioral data gathered outside of achievement contexts—specifically, natural music-listening behavior. Using a multimethod design that combined psychometric assessment with five months of mobile-sensing data, both administered on smartphones, we extracted audio- and lyrics-based music-preference features, along with habitual listening behaviors, as predictors of GCA in LASSO and random forest models. Subsequently, we evaluated the contribution of different feature groups and individual features to the models’ performance.

Our findings show that everyday music-listening behavior contains some, though limited, predictive information about individuals’ GCA. In particular, only the nonlinear random forest model detected meaningful patterns in the data, yielding modest out-of-sample predictive performance and suggesting that associations between music-listening behavior and GCA may be weak, nonlinear, piecewise, or feature-interaction dependent, rather than following simple additive linear relationships. Nevertheless, the results offer initial evidence that combining mobile sensing with machine learning can reveal detectable associations between natural-listening behavior and cognitive ability. Importantly, different types of features contributed unequally to prediction performance. Preferences for song lyrics—including features such as present-focus, social word use, and positive emotional tone—emerged as the most informative group of predictors, followed by listening habits (e.g., overall listening duration). In contrast, audio preferences played a comparatively minor role, with only the liveness of recordings showing notable relevance.

In the following sections, we interpret these findings in relation to existing literature and theory, outline key limitations of this approach, and provide suggestions for future research.

### 4.1. Predicting GCA from Music-Listening Behavior

Although predictive performance was modest overall, the results indicate that the random forest model was able to detect some meaningful signal in the music-listening data (*ρ_md_* = 0.10). While these findings may appear disappointing at first glance, they are reasonable when considered in light of the relatively small sample size and the broader literature. For instance, a meta-analysis on the predictability of individual differences from digital data reported an average correlation of *r* = 0.29 for intelligence in studies using social media behavior (Settanni et al., 2018), which captures a far wider range of behaviors and may even indirectly reflect musical preferences (e.g., following or posting about artists). Likewise, even human self-judgment (*r* = 0.30; Freund & Kasten, 2012) and other-judgment of cognitive abilities (*r* = 0.43; Machts et al., 2016) achieved higher, yet still not optimal, meta-analytic correlations in the past. Against these benchmarks, the limited predictive value of music listening alone, a purely leisure activity disconnected from performance in achievement or everyday settings, appears quite plausible. 

Turning back to music-listening behavior itself, it is notable that the personality trait Openness, which is commonly linked to intelligence (Anglim et al., 2022; DeYoung, 2015), has been predicted more accurately from listening patterns in previous machine learning work (I. Anderson et al., 2020; Sust et al., 2023). This suggests that music-listening behavior contains psychological information beyond intellectual capabilities. Moreover, prior studies examining cognitive abilities and music preferences have typically reported only small associations, often based on self-reports, even smaller samples, and in-sample estimations (Bonetti & Costa, 2016; George et al., 2007; Kanazawa & Perina, 2012; Pirgon, 2021; Račevska & Tadinac, 2019; Rentfrow & Gosling, 2003). Our generalization to the real world aligns with this literature, reinforcing the view that the relationship between music listening and intelligence is subtle but detectable.

Beyond assessing the overall predictability of GCA from music-listening behavior, we also explored which aspects of listening behavior carried predictive signal. Our analyses revealed meaningful differences across feature groups and individual features, offering insights into how cognitive ability may subtly manifest in naturalistic music consumption. 

Overall, lyrics-based preferences emerged as the strongest predictors of GCA, followed by listening habits, whereas audio-based preferences contributed comparatively little. The differential importance of audio versus lyrics preferences makes sense given that melodic and lyrical components are independently processed in the cognition system (Besson et al., 1998; Bonnel et al., 2001). The prominence of lyrics-based preferences is somewhat surprising in light of previous work showing that audio features tend to be more informative for predicting the personality trait Openness (Qiu et al., 2019; Sust et al., 2023). However, the pattern is intuitively interpretable, given that comprehension knowledge is one of the domains represented in our GCA measure. Lyrics, by virtue of their linguistic content, may be especially relevant for capturing variance in verbal or knowledge-related abilities. Melodic features may be more strongly related to other cognitive domains, such as auditory processing, than to the broader construct of GCA (Schneider & McGrew, 2018). The relatively low relevance of audio-based preferences also contrasts with earlier work linking preferences for melodically complex or instrumental genres (e.g., blues, classical) to cognitive ability (Račevska & Tadinac, 2019; Rentfrow & Gosling, 2003). This discrepancy may reflect a gap between self-reported genre preferences and behavioral listening data: what people report enjoying may not fully align with what they actually listen to in daily life (Greenberg & Rentfrow, 2017). Our results imply that once real-world behavior is examined, lyrical content may capture more cognitively relevant variance than melodic complexity alone.

At the individual feature level, several patterns offer additional insight. First, variances in musical preferences, often associated with Openness and cognitively stimulating behavior (Bansal et al., 2021), were not systematically among the most predictive features. This suggests that cognitive ability may manifest in specific listening tendencies rather than in the breadth or diversity of musical taste. The most influential audio feature for predicting GCA was lower liveness, indicating a preference for recordings without audible audience presence. One possible interpretation for this finding draws on Uses and Gratifications theory (DeNora, 2000; Katz et al., 1973): Live recordings are typically more energetic, dynamic, and less controlled, which may make them less suitable for cognitively oriented uses of music, such as focused listening or analysis. Such cognitive music use has previously been found to be particularly relevant among those with better cognitive functioning (Bonetti et al., 2021; Chamorro-Premuzic & Furnham, 2007). Similar considerations may also apply to the lyric-related features that proved informative in our analyses. Preferences for lyrics with a less positive emotional tone are consistent with prior research showing that sad or melancholic music can facilitate introspection and life reflection (Saarikallio & Erkkilä, 2007), both of which are rather cognitive uses of music. Conversely, lyrics characterized by lower social word use, greater authenticity, and more home-related content may indicate a focus on personal meaning-making rather than social engagement, aligning with findings that more intelligent individuals tend to use music less for social or emotional purposes (Bonetti et al., 2021; Chamorro-Premuzic & Furnham, 2007). Likewise, less tentative and more present-focused lyrics may appeal to listeners who prefer clearer, more straightforward messages—potentially reflecting more decisive or analytically oriented engagement with music. The predictive relevance of German-language music may reflect individual differences in verbal abilities or experiences beyond the native language (i.e., foreign language proficiency), which are captured by the GCA measure. Future studies that assess more specific (verbal) abilities of the Cattell–Horn–Carroll model may help clarify this association (Schneider & McGrew, 2018). Finally, the finding that longer listening durations were associated with higher predicted GCA may indicate that individuals with higher cognitive ability may use music more consistently throughout their routines or simply engage more with (digital) media. However, all of these interpretation attempts are purely speculative given our exploratory study design, modest predictive performance, potentially nonlinear effects, and the lack of external model validation on an independent dataset. Further research is needed to confirm feature-level associations with GCA before assigning them a stronger theoretical meaning. That is especially true for the lyrics-related features, as past studies have shown that LIWC categories yield only small effect sizes and have limited generalizability, at least in personality prediction contexts (Koutsoumpis et al., 2022; Martínez-Huertas et al., 2022).

Beyond the identified feature patterns, music listening may also relate to GCA through adaptive music selection. If GCA reflects the capacity to adapt effectively to one’s environment (Gottfredson, 1997; Stasielowicz, 2020), then choosing music that fits situational demands may itself be an adaptive behavior. Prior research shows that people adjust their music choices in accordance with momentary states and contextual factors, including mood (Taruffi & Koelsch, 2014; Randall & Rickard, 2017) and situational constraints (Greb et al., 2018; Park et al., 2019). Thus, some links between music listening and GCA may stem from individuals’ ability to flexibly tailor their musical environments to ongoing situational demands, rendering music more than a mere leisure activity.

### 4.2. Opportunities and Challenges When Predicting GCA

Combining digital behavioral data with state-of-the-art machine learning offers a promising avenue for uncovering subtle and potentially complex indicators of individual differences, as has been repeatedly suggested in personality research (Peters et al., 2022; Phan & Rauthmann, 2021). Although GCA is traditionally assessed through formal tests administered in controlled laboratory or achievement-oriented settings (Flanagan & McDonough, 2018; Koch et al., 2021), cognitive abilities also manifest in everyday behavior (Gottfredson, 1997; Schultheis & Cooper, 2022). Extending this logic to digital trace data, demonstrating that GCA can be inferred—even modestly—from naturalistic behavioral patterns would enable low-threshold, ecologically valid proxies for cognitive assessment and pave the way for adaptive digital environments or early intervention strategies (Koch et al., 2021). Studies such as ours, which test both the potential and the limitations of using digital behavior to infer cognitive ability, may help stimulate innovative approaches for assessing human behavior in situ (see Bergmann et al., 2025). In the long run, such methods could support individualized digital experiences (e.g., tailored music recommendations, interface adaptations) or even intervention systems (e.g., referral to clinical assessment) that respond to early signs of cognitive decline. At the same time, these possibilities underscore the importance of addressing ethical, transparent, and user-centered practices in the collection and application of digital behavioral data. While our study highlights the potential of digital behavioral data for predicting cognitive abilities, several conceptual and methodological challenges must be addressed before such approaches can be applied more successfully and broadly.

First, determining an appropriate ground truth for model training is nontrivial. Predictive modeling requires a reliable and theoretically justified measure of cognitive ability, yet intelligence itself is a multidimensional construct with ongoing debates about its structure (Kovacs & Conway, 2019). We relied on a general cognitive ability score derived from three subtests of the Cattell–Horn–Carroll theory, which is widely accepted but encompasses far more facets than those assessed here (Schneider & McGrew, 2018; Schuhfried, 2022). It remains unclear which aspects of intelligence are most amenable to prediction from specific behaviors like music listening: the general factor or more specific secondary factors, such as fluid or crystallized intelligence, or even auditory processing ability (Horn & Cattell, 1966; McGrew, 2005). This is equivalent to the question whether prediction efforts should target narrow domains more closely aligned with the behavior of interest or broad factors meaningful for general life outcomes (e.g., Deary et al., 2021; Roth et al., 2015; Schmidt & Hunter, 2004). A related challenge concerns the type of cognitive assessment used as ground truth. Traditional supervised tests administered in controlled settings (Flanagan & McDonough, 2018) offer high standardization but are time-consuming and hinder the collection of large datasets needed for machine learning (see the third issue). More flexible digital assessments have been proposed (Koch et al., 2021), and we used a validated smartphone-based test (Schuhfried, 2022). However, mobile tests may be more vulnerable to careless responding or environmental distractions. To alleviate this risk, emerging gamified assessments could increase engagement (Foroughi et al., 2016; Kokkinakis et al., 2017). Anyways, the quality of prediction models will always depend on the validity of the underlying ground truth, warranting careful consideration of assessment strategy.

Second, digital behavioral data themselves contain substantial noise. Although often regarded as objective, behavioral traces do not always reflect the participant’s own preferences or actions. For instance, it remains unclear to what extent music played on smartphone streaming apps reflects active selection versus passive exposure through playlists or algorithmic recommendations, introducing noise into preference measures while still reflecting music-listening behavior as it occurs naturally. Moreover, digital traces typically require enrichment (see Schoedel et al., 2025), such as mapping songs to audio or lyric features, which may introduce missingness or measurement error. In our case, not all songs could be matched to audio or lyrical metadata, and our closed-vocabulary language-processing tool, while interpretable, was limited by dictionary coverage (Eichstaedt et al., 2021). Although such features do not aim to measure latent constructs in the psychometric sense, reliability and validity considerations remain pertinent for sensing-based data pipelines.

Third, suitable sample sizes and sensing coverage are essential for building robust models. Machine learning models typically require large datasets with substantial behavioral variability to learn stable, generalizable patterns (Peters et al., 2022). Our final sample was considerably reduced because only 185 of the 380 participants with GCA data listened to music on their smartphones. Some participants may not have listened to music at all, while others may have relied on other devices such as laptops or smart speakers. Fully capturing everyday behaviors, therefore, requires sensing across multiple devices or platforms (e.g., I. Anderson et al., 2020). This concern is mitigated when attention is not focused on a specific behavior, such as music listening, but rather on heterogeneous behavioral patterns (Bergmann et al., 2025). As everyday actions increasingly leave digital traces (Boyd et al., 2020; Renner et al., 2020), future research can draw on a wider array of behaviors beyond smartphone-based music listening (e.g., app usage, mobility patterns; Schoedel et al., 2023; Stachl et al., 2020a).

Fourth, generalizability remains a key concern. Despite using cross-validation, it is unclear whether our model generalizes to populations with different demographic, cultural, or technological characteristics. Although our sample originated from a quota-based recruitment procedure and included a mix of ages and education levels, sampling biases may persist (for more details on our sampling procedure, see Schoedel et al., 2023 ). For example, music preferences vary across cultures (Bello & Garcia, 2021; Park et al., 2019), and our focus on Android users may introduce educational or socioeconomic differences (Keusch et al., 2020; Schoedel et al., 2026). In addition, generalizability across time may be challenged by concept drift, that is, systematic changes in behavioral patterns as individuals adopt new platforms and devices (Stachl et al., 2020b) or as musical styles and listening practices evolve at the societal level (IFPI, 2023). Future work should test models on larger, more diverse samples and over time.

Finally, it is important to emphasize that machine learning approaches applied to digital trace data capture correlational associations rather than causal relationships (Pargent et al., 2023). Accordingly, the present findings do not permit conclusions about whether music-listening behavior reflects underlying cognitive abilities, is influenced by them, or even shapes them, nor do they permit conclusions about whether the observed associations are driven by unobserved third variables. For example, both cognitive ability and music preferences were associated with age in our sample (see Descriptive Statistics and Table S2), raising the possibility that the models learned age-related patterns rather than direct links between music listening and cognitive ability. Consequently, research aiming to assess cognitive abilities using digital behavioral data should carefully control for potential confounds and cannot, without further experimental or longitudinal evidence, justify prescriptive applications based on the observed associations.

These five challenges underscore that more conceptual and empirical work is necessary before inferring cognitive abilities from digital behavioral data at scale.

## 5. Conclusions

The present study provides an early exploration of whether general cognitive ability can be inferred from digital traces of non-achievement-related, everyday behaviors, specifically, natural music-listening patterns captured through mobile sensing on smartphones. Although predictive performance was modest, our findings nonetheless offer first indications that everyday listening behavior contains detectable signal, particularly through lyrical preferences and certain listening habits. These results extend existing work by demonstrating that intelligence, typically assessed through structured performance tests, may also leave subtle traces in routine behaviors outside of formal achievement contexts. Our findings further suggest that digital behavioral data could, in principle, complement traditional cognitive assessments but also highlight key challenges that must be addressed before such methods can be meaningfully deployed.

## Figures and Tables

**Table 1:** Top 10 most important features for random forest models.

Feature	Group	*ρ*-loss_mdn_	*r*
average liveness	audio preferences	−0.0166	−0.20
average social processes	lyrics preferences	−0.0155	−0.23
average present focus	lyrics preferences	−0.0132	0.28
percentage of German songs	listening habits	−0.0080	−0.23
variance social processes	lyrics preferences	−0.0070	−0.07
average authenticity	lyrics preferences	−0.0063	0.22
average tentative words	lyrics preferences	−0.0062	−0.17
average positive tone	lyrics preferences	−0.0060	−0.17
average home words	lyrics preferences	−0.0059	0.03
average listening duration	listening habits	−0.0047	0.18

Note. The top 10 features are presented in decreasing order of median permutation-based feature importance in terms of *ρ*-loss across the 100 resampling iterations. Importance scores for each iteration are based on median *ρ*-loss across 10 random permutations. *r* = Pearson correlation coefficient between the respective feature and the GCA scores in the full sample.

## Data Availability

While we cannot share raw mobile-sensing data due to privacy reasons, aggregated data and all analysis scripts used in this project are openly available in our repository on the OSF: https://osf.io/cz4dj/ (accessed on 6 February 2026).

## Supplementary Materials

The following supporting information can be downloaded at https://www.mdpi.com/article/10.3390/jintelligence14020029/s1 (see also https://osf.io/cz4dj/ (accessed on 6 February 2026)), Table S1: Descriptive Statistics for the 215 Music-Listening Features and their Pearson Correlations with GCA; Table S2: Pearson Correlations Between the 215 Music-Listening Features and the Demographic Variables; Table S3: Permutation-Based Feature Importances for the Random Forest Models.

## Abbreviations

The following abbreviations are used in this manuscript:

App	Application
GCA	General Cognitive Ability
INT	Inventory for Testing Cognitive Abilities
LIWC	Linguistic Inquiry and Word Count
MSE	Mean Squared Error

## Appendix A. Supplementary Results

**Table A1:** Prediction performance across resampling iterations by model.

	*ρ*					MSE				
	*Mdn*	*q*1	*q*3	*M*	*SD*	*Mdn*	*q*1	*q*3	*M*	*SD*
LASSO	0.00	0.00	0.15	0.06	0.14	17.30	15.64	19.38	17.52	3.14
Random Forest	0.10	0.00	0.22	0.11	0.16	17.04	15.46	19.12	17.28	2.99
Baseline	0.00	0.00	0.00	0.00	0.00	17.21	15.50	18.70	17.17	2.97

Note. Aggregated out-of-sample prediction performance for GCA by model and performance metric across test folds in 20-times repeated five-fold cross-validation. The coefficient *ρ* indicates the association between measured and predicted GCA scores. The Baseline indicates the performance of a featureless model. LASSO = least absolute shrinkage and selection operator regression. *q*1 = 1st quartile, *q*3 = 3rd quartile.

**Table A2:** Grouped feature importance.

	Importance				
Feature Group	*Mdn*	*q*1	*q*3	Rank	*n_feat_*
Audio Preferences	0.003	−0.032	0.030	3	32
Lyrics Preferences	−0.057	−0.150	0.056	1	148
Listening Habits	−0.020	−0.062	0.003	2	17

Note. Aggregated grouped features importance in terms of median out-of-sample *ρ*-loss across 20-times repeated five-fold cross-validation. *ρ*-loss per test fold is based on 10 random permutations. *q*1 = 1st quartile, *q*3 = 3rd quartile, *n*feat = number of features from each group after target-independent preprocessing.
