# Supplementary material for: Deep Beats, Deep Thoughts? Predicting General Cognitive Ability from Natural Music-Listening Behavior
Source: J Intell. 2026 Feb 13;14(2):29. doi: 10.3390/jintelligence14020029 (PMC12941781; doi:10.3390/jintelligence14020029)
Supplement: Supplementary file 1 [file jintelligence-14-00029-s001.zip › jintelligence-4046931-supplementary.pdf]

Table S1

Descriptive Statistics for the 215 Music-Listening Features and their Pearson Correlations with GCA

Feature	*M*	*SD*	Min	Max	r_{GCA}
audio_acousticness_avg	0.16	0.18	0.00	0.97	0.10
audio_acousticness_var	0.14	0.14	0.00	0.58	0.00
audio_danceability_avg	0.60	0.10	0.30	0.88	-0.04
audio_danceability_var	0.11	0.06	0.00	0.22	0.01
audio_energy_avg	0.70	0.15	0.04	0.99	-0.17
audio_energy_var	0.15	0.09	0.00	0.51	0.06
audio_instrumentalness_avg	0.03	0.13	0.00	0.90	0.08
audio_instrumentalness_var	0.01	0.07	0.00	0.60	0.03
audio_key_0	9.57	10.48	0.00	100.00	0.18
audio_key_1	8.03	10.20	0.00	100.00	0.13
audio_key_2	8.17	10.27	0.00	100.00	0.04
audio_key_3	2.79	7.86	0.00	100.00	-0.04
audio_key_4	6.66	9.92	0.00	100.00	-0.03
audio_key_5	7.86	14.22	0.00	100.00	-0.08
audio_key_6	5.17	4.75	0.00	26.32	-0.02
audio_key_7	11.79	17.44	0.00	100.00	-0.03
audio_key_8	4.42	4.13	0.00	20.00	0.05
audio_key_9	8.99	9.95	0.00	64.29	-0.09
audio_key_10	6.34	13.76	0.00	100.00	-0.02
audio_key_11	7.23	10.86	0.00	100.00	-0.03
audio_liveness_avg	0.15	0.09	0.03	0.91	-0.20
audio_liveness_var	0.07	0.06	0.00	0.36	-0.20
audio_loudness_avg	-6.94	2.47	-25.03	-1.50	-0.13
audio_loudness_var	2.07	1.36	0.00	8.31	0.06
audio_matches_perc	0.85	0.19	0.00	1.00	0.00
audio_mode_1	63.71	21.41	0.00	100.00	0.06
audio_speechiness_avg	0.06	0.04	0.02	0.29	-0.11
audio_speechiness_var	0.02	0.03	0.00	0.18	-0.09
audio_tempo_avg	120.93	14.74	81.47	205.53	-0.05
audio_tempo_var	21.69	12.98	0.00	72.53	0.08
audio_valence_avg	0.49	0.17	0.06	0.97	-0.12
audio_valence_var	0.20	0.11	0.00	0.50	-0.03
lyrics_Analytic_avg	26.44	16.43	1.00	88.53	-0.01
lyrics_Analytic_var	23.49	15.08	0.00	62.14	-0.04
lyrics_Authentic_avg	79.44	15.49	12.29	99.00	0.22
lyrics_Authentic_var	21.23	15.44	0.00	69.36	-0.16
lyrics_Clout_avg	69.14	15.68	1.00	98.72	-0.08
lyrics_Clout_var	26.92	14.34	0.00	62.35	0.05
lyrics_Dic_avg	90.31	3.57	72.48	98.08	0.11
lyrics_Dic_var	5.20	3.29	0.00	24.43	-0.04
lyrics_Sixltr_avg	9.28	2.68	3.08	20.90	-0.14

lyrics_Sixltr_var	4.15	2.38	0.00	14.90	-0.03
lyrics_Tone_avg	47.28	22.83	1.00	99.00	-0.17
lyrics_Tone_var	34.66	18.69	0.00	65.56	0.01
lyrics_WC_avg	296.30	81.53	156.00	795.00	-0.01
lyrics_WC_var	88.21	53.82	0.00	361.75	0.06
lyrics_WPS_avg	146.82	66.52	29.33	459.00	0.02
lyrics_WPS_var	101.43	53.48	0.00	220.31	-0.06
lyrics_achieve_avg	0.96	0.91	0.00	8.31	-0.05
lyrics_achieve_var	0.92	0.59	0.00	2.73	-0.05
lyrics_adj_avg	3.92	1.48	1.59	12.06	-0.06
lyrics_adj_var	2.20	1.19	0.00	8.38	-0.06
lyrics_adverb_avg	4.89	1.65	0.00	13.49	0.01
lyrics_adverb_var	2.44	1.19	0.00	4.59	-0.02
lyrics_affect_avg	5.72	1.84	1.59	15.34	-0.10
lyrics_affect_var	2.71	1.33	0.00	5.32	-0.05
lyrics_affiliation_avg	2.19	1.43	0.00	9.71	-0.10
lyrics_affiliation_var	2.00	1.11	0.00	5.68	-0.05
lyrics_anger_avg	0.25	0.35	0.00	2.56	-0.08
lyrics_anger_var	0.27	0.37	0.00	1.78	0.01
lyrics_anx_avg	0.11	0.55	0.00	6.25	-0.07
lyrics_anx_var	0.04	0.15	0.00	0.89	0.03
lyrics_article_avg	5.40	1.90	0.00	17.06	0.04
lyrics_article_var	2.65	1.34	0.00	5.86	0.02
lyrics_assent_avg	0.36	0.88	0.00	7.99	-0.12
lyrics_assent_var	0.30	0.54	0.00	3.40	-0.02
lyrics_auxverb_avg	9.78	2.21	3.49	23.05	0.16
lyrics_auxverb_var	3.51	1.67	0.00	7.41	0.06
lyrics_bio_avg	3.29	1.39	0.31	10.42	-0.19
lyrics_bio_var	2.09	1.16	0.00	8.20	-0.11
lyrics_body_avg	1.12	0.88	0.00	10.07	-0.07
lyrics_body_var	0.98	0.50	0.00	2.51	0.06
lyrics_cause_avg	1.21	0.58	0.00	4.39	0.01
lyrics_cause_var	1.02	0.56	0.00	2.97	0.06
lyrics_certain_avg	2.43	1.40	0.00	10.83	-0.10
lyrics_certain_var	1.75	0.93	0.00	4.78	-0.08
lyrics_cogproc_avg	11.69	3.00	4.68	27.50	-0.14
lyrics_cogproc_var	4.61	2.23	0.00	11.00	0.06
lyrics_compare_avg	1.50	1.07	0.34	11.26	-0.02
lyrics_compare_var	1.19	0.86	0.00	9.27	-0.04
lyrics_conj_avg	6.34	2.55	1.34	18.68	-0.13
lyrics_conj_var	2.95	1.65	0.00	7.55	-0.04
lyrics_death_avg	0.11	0.60	0.00	6.47	0.06
lyrics_death_var	0.07	0.42	0.00	4.34	-0.13
lyrics_differ_avg	2.39	1.24	0.00	9.38	-0.10

lyrics_differ_var	1.59	0.80	0.00	3.88	0.04
lyrics_discrep_avg	1.53	0.85	0.00	5.81	-0.14
lyrics_discrep_var	1.33	0.73	0.00	3.89	0.00
lyrics_drives_avg	7.06	1.74	0.30	17.76	0.03
lyrics_drives_var	3.37	1.69	0.00	9.21	-0.01
lyrics_family_avg	0.12	0.33	0.00	2.41	-0.17
lyrics_family_var	0.12	0.37	0.00	3.57	-0.09
lyrics_feel_avg	0.75	0.64	0.00	6.05	0.05
lyrics_feel_var	0.81	0.70	0.00	7.69	0.06
lyrics_female_avg	0.28	1.04	0.00	7.56	-0.02
lyrics_female_var	0.16	0.94	0.00	11.21	-0.04
lyrics_filler_avg	0.05	0.45	0.00	5.56	-0.15
lyrics_filler_var	0.01	0.09	0.00	0.85	-0.11
lyrics_focusfuture_avg	1.48	0.90	0.00	5.88	0.15
lyrics_focusfuture_var	1.33	0.74	0.00	3.72	0.11
lyrics_focuspast_avg	2.13	1.45	0.00	10.59	-0.06
lyrics_focuspast_var	1.74	1.03	0.00	7.78	0.02
lyrics_focuspresent_avg	13.98	2.89	4.03	24.85	0.28
lyrics_focuspresent_var	4.57	2.24	0.00	10.57	0.04
lyrics_friend_avg	0.06	0.21	0.00	1.47	0.03
lyrics_friend_var	0.02	0.08	0.00	0.61	0.09
lyrics_function._avg	55.86	4.28	36.81	67.07	0.11
lyrics_function._var	6.37	3.27	0.00	14.44	0.03
lyrics_health_avg	0.39	0.60	0.00	6.97	0.05
lyrics_health_var	0.37	0.31	0.00	1.56	0.01
lyrics_hear_avg	0.76	0.55	0.00	4.74	-0.05
lyrics_hear_var	0.80	0.49	0.00	2.79	-0.03
lyrics_home_avg	0.09	0.39	0.00	4.28	0.03
lyrics_home_var	0.04	0.13	0.00	0.93	-0.20
lyrics_i_avg	8.09	3.00	0.00	24.55	0.09
lyrics_i_var	4.79	2.39	0.00	12.02	-0.02
lyrics_informal_avg	2.87	1.93	0.00	16.32	-0.13
lyrics_informal_var	2.45	1.47	0.00	7.07	0.00
lyrics_ingest_avg	0.08	0.22	0.00	1.18	-0.09
lyrics_ingest_var	0.05	0.15	0.00	0.77	-0.06
lyrics_insight_avg	1.86	0.79	0.00	5.43	-0.02
lyrics_insight_var	1.31	0.66	0.00	3.85	0.08
lyrics_interrog_avg	1.41	0.59	0.00	5.11	-0.07
lyrics_interrog_var	1.16	0.59	0.00	2.65	0.00
lyrics_ipron_avg	4.55	1.26	0.50	11.02	0.11
lyrics_ipron_var	2.51	1.22	0.00	5.93	-0.05
lyrics_leisure_avg	0.82	0.75	0.00	5.47	-0.16
lyrics_leisure_var	0.82	0.73	0.00	6.26	-0.15
lyrics_male_avg	0.21	0.77	0.00	4.78	-0.04

lyrics_male_var	0.10	0.34	0.00	2.39	-0.02
lyrics_matches_perc	0.60	0.23	0.00	1.00	0.04
lyrics_money_avg	0.09	0.24	0.00	1.13	0.00
lyrics_money_var	0.06	0.18	0.00	1.25	-0.08
lyrics_motion_avg	2.72	1.33	0.60	13.94	0.08
lyrics_motion_var	1.71	0.90	0.00	4.45	0.05
lyrics_negate_avg	2.51	1.89	0.54	22.75	0.14
lyrics_negate_var	1.79	1.01	0.00	7.28	-0.01
lyrics_negemo_avg	1.84	1.03	0.00	10.51	0.02
lyrics_negemo_var	1.41	0.74	0.00	3.41	0.11
lyrics_netspeak_avg	0.21	0.46	0.00	4.36	0.02
lyrics_netspeak_var	0.21	0.38	0.00	2.68	-0.03
lyrics_nonflu_avg	0.30	0.59	0.00	4.76	0.04
lyrics_nonflu_var	0.27	0.42	0.00	2.92	0.11
lyrics_number_avg	0.40	0.43	0.00	4.12	-0.06
lyrics_number_var	0.42	0.30	0.00	1.25	-0.02
lyrics_percept_avg	3.64	1.29	0.00	10.90	0.06
lyrics_percept_var	2.10	1.24	0.00	8.79	-0.04
lyrics_posemo_avg	3.25	1.61	0.86	12.18	-0.09
lyrics_posemo_var	2.01	1.09	0.00	6.92	-0.05
lyrics_power_avg	1.59	1.16	0.00	9.87	0.14
lyrics_power_var	1.26	0.74	0.00	6.13	0.04
lyrics_ppron_avg	16.31	3.18	8.24	31.82	0.05
lyrics_ppron_var	4.88	2.39	0.00	10.17	-0.05
lyrics_prep_avg	9.98	1.99	4.51	24.67	0.11
lyrics_prep_var	3.08	1.62	0.00	5.90	0.03
lyrics_pronoun_avg	21.78	3.05	11.76	35.23	0.05
lyrics_pronoun_var	5.24	2.51	0.00	10.69	-0.01
lyrics_quant_avg	1.76	1.07	0.00	7.74	-0.14
lyrics_quant_var	1.35	0.86	0.00	7.05	-0.08
lyrics_relativ_avg	16.02	3.11	2.99	33.77	0.08
lyrics_relativ_var	5.14	2.45	0.00	11.12	-0.05
lyrics_relig_avg	0.20	0.65	0.00	4.64	0.10
lyrics_relig_var	0.21	0.75	0.00	5.74	-0.01
lyrics_reward_avg	0.87	0.83	0.00	8.04	0.01
lyrics_reward_var	0.84	0.54	0.00	2.92	0.07
lyrics_risk_avg	0.23	0.33	0.00	2.43	-0.06
lyrics_risk_var	0.24	0.23	0.00	1.19	0.07
lyrics_sad_avg	0.48	0.40	0.00	2.96	0.04
lyrics_sad_var	0.50	0.34	0.00	1.38	0.05
lyrics_see_avg	1.18	0.80	0.00	6.54	0.13
lyrics_see_var	1.01	0.52	0.00	2.74	0.07
lyrics_sexual_avg	0.11	0.53	0.00	6.25	-0.14
lyrics_sexual_var	0.07	0.21	0.00	1.31	-0.05

lyrics_shehe_avg	0.47	1.08	0.00	5.85	-0.07
lyrics_shehe_var	0.29	0.77	0.00	7.18	-0.12
lyrics_social_avg	13.55	3.01	5.15	27.27	-0.23
lyrics_social_var	5.18	2.58	0.00	12.51	-0.07
lyrics_space_avg	7.53	2.24	1.14	18.75	0.08
lyrics_space_var	3.11	1.61	0.00	5.92	0.03
lyrics_swear_avg	0.05	0.18	0.00	1.41	-0.08
lyrics_swear_var	0.03	0.16	0.00	1.11	-0.01
lyrics_tentat_avg	1.51	0.75	0.00	5.88	-0.17
lyrics_tentat_var	1.27	0.66	0.00	2.80	0.02
lyrics_they_avg	0.13	0.39	0.00	2.84	-0.11
lyrics_they_var	0.10	0.27	0.00	2.08	-0.17
lyrics_time_avg	5.28	1.79	0.30	15.69	0.06
lyrics_time_var	2.72	1.36	0.00	6.67	-0.04
lyrics_verb_avg	19.99	2.63	11.33	31.10	0.15
lyrics_verb_var	4.73	2.49	0.00	12.13	0.08
lyrics_we_avg	0.75	1.21	0.00	8.74	0.01
lyrics_we_var	0.70	0.88	0.00	6.57	-0.04
lyrics_work_avg	0.18	0.29	0.00	1.52	-0.15
lyrics_work_var	0.15	0.26	0.00	1.08	-0.07
lyrics_you_avg	4.33	1.85	0.00	11.76	0.00
lyrics_you_var	3.45	1.72	0.00	8.50	0.05
habits_daysMusic_perc	0.32	0.30	0.00	1.00	0.13
habits_songs_num	696.51	1199.34	0.00	7356.00	0.12
habits_songs_DE_perc	0.23	0.28	0.00	1.00	-0.23
habits_songs_EN_perc	0.70	0.30	0.00	1.00	0.13
habits_songs_unique_language_num	3.93	3.61	0.00	29.00	0.12
habits_songs_duration_avg	3.36	1.20	0.00	11.59	0.18
habits_songs_duration_var	0.92	0.65	0.00	3.58	-0.04
habits_skipped_songs_num	309.29	738.88	0.00	4998.00	0.11
habits_skipped_songs_duration_avg	0.05	0.06	0.00	0.26	-0.08
habits_skipped_songs_duration_var	0.04	0.04	0.00	0.18	0.05
habits_unique_songs_num	323.76	456.55	1.00	2481.00	0.05
habits_unique_artists_num	152.87	203.74	1.00	1036.00	0.09
habits_unique_albums_num	211.67	306.00	1.00	1802.00	0.10
habits_musicapp_totalPerDay_num_avg	2.99	2.45	1.00	19.00	-0.06
habits_musicapp_totalPerDay_num_var	1.98	1.89	0.00	11.12	-0.04
habits_musicapp_totalPerDay_duration_avg	3.53	5.13	0.03	42.08	-0.12
habits_musicapp_totalPerDay_duration_var	3.69	5.81	0.00	47.33	-0.16
habits_unique_musicapps_num	1.97	1.07	1.00	6.00	-0.18
habits_musicapp_session_duration_avg	0.65	0.66	0.03	6.34	-0.05
habits_musicapp_session_duration_var	0.65	0.67	0.00	4.39	-0.08

Table S2

Pearson Correlations Between the 215 Music-Listening Features and the Demographic Variables

	r		
Feature	Age	Gender	Education
audio_acousticness_avg	0.16	0.13	0.12
audio_acousticness_var	0.00	0.23	-0.04
audio_danceability_avg	-0.33	0.20	-0.07
audio_danceability_var	0.00	0.05	0.00
audio_energy_avg	-0.18	-0.19	-0.07
audio_energy_var	-0.01	0.09	-0.04
audio_instrumentalness_avg	0.11	-0.01	0.08
audio_instrumentalness_var	0.05	0.01	0.09
audio_key_0	-0.03	0.09	0.11
audio_key_1	-0.26	0.02	0.00
audio_key_2	0.09	0.08	-0.07
audio_key_3	0.03	-0.03	0.07
audio_key_4	0.09	-0.02	-0.05
audio_key_5	-0.15	0.02	0.08
audio_key_6	-0.24	0.13	-0.09
audio_key_7	0.09	-0.1	0.03
audio_key_8	-0.23	0.11	0.06
audio_key_9	-0.09	0.00	-0.03
audio_key_10	-0.01	-0.15	-0.02
audio_key_11	-0.04	0.01	0.05
audio_liveness_avg	0.13	-0.11	-0.16
audio_liveness_var	0.09	-0.05	-0.20
audio_loudness_avg	-0.28	-0.02	-0.12
audio_loudness_var	0.13	0.03	0.01
audio_matches_perc	0.08	0.04	-0.08
audio_mode_1	0.10	-0.04	0.02
audio_speechiness_avg	-0.04	0.09	-0.05
audio_speechiness_var	-0.26	0.11	-0.05
audio_tempo_avg	-0.16	-0.16	-0.04
audio_tempo_var	-0.05	0.06	0.00
audio_valence_avg	-0.16	-0.01	0.10
audio_valence_var	-0.09	0.03	0.01
lyrics_Analytic_avg	0.13	-0.06	0.00
lyrics_Analytic_var	0.07	-0.05	0.06
lyrics_Authentic_avg	-0.13	0.14	0.21
lyrics_Authentic_var	0.05	0.01	-0.17
lyrics_Clout_avg	0.26	-0.04	-0.07
lyrics_Clout_var	-0.19	0.09	-0.03
lyrics_Dic_avg	0.03	0.03	0.08
lyrics_Dic_var	-0.13	0.08	-0.02

lyrics_Sixltr_avg	0.13	-0.09	0.00
lyrics_Sixltr_var	-0.02	0.07	0.06
lyrics_Tone_avg	0.21	0.05	-0.16
lyrics_Tone_var	-0.12	0.13	0.02
lyrics_WC_avg	-0.33	0.21	-0.05
lyrics_WC_var	-0.23	0.13	-0.01
lyrics_WPS_avg	-0.16	0.08	0.01
lyrics_WPS_var	-0.27	0.26	-0.02
lyrics_achieve_avg	0.01	0.09	-0.11
lyrics_achieve_var	0.01	0.04	0.00
lyrics_adj_avg	0.09	-0.05	0.04
lyrics_adj_var	0.10	0.03	-0.02
lyrics_adverb_avg	-0.06	-0.07	0.18
lyrics_adverb_var	-0.05	0.05	0.00
lyrics_affect_avg	0.13	-0.11	-0.11
lyrics_affect_var	-0.11	0.04	-0.08
lyrics_affiliation_avg	0.02	-0.08	0.14
lyrics_affiliation_var	-0.07	0.10	0.01
lyrics_anger_avg	-0.09	-0.05	0.07
lyrics_anger_var	-0.11	0.03	0.06
lyrics_anx_avg	-0.03	-0.13	0.07
lyrics_anx_var	0.23	0.04	-0.19
lyrics_article_avg	0.17	-0.03	0.04
lyrics_article_var	-0.01	0.07	0.10
lyrics_assent_avg	0.12	0.01	-0.09
lyrics_assent_var	-0.07	0.24	-0.21
lyrics_auxverb_avg	-0.08	-0.09	0.10
lyrics_auxverb_var	-0.09	0.06	-0.04
lyrics_bio_avg	0.15	-0.04	-0.08
lyrics_bio_var	-0.04	0.02	-0.06
lyrics_body_avg	0.19	-0.03	-0.09
lyrics_body_var	-0.05	0.04	0.00
lyrics_cause_avg	-0.16	0.14	0.00
lyrics_cause_var	-0.16	0.15	-0.02
lyrics_certain_avg	0.06	0.01	-0.11
lyrics_certain_var	-0.04	-0.01	-0.06
lyrics_cogproc_avg	-0.05	0.06	-0.06
lyrics_cogproc_var	-0.08	0.12	0.05
lyrics_compare_avg	-0.03	0.07	-0.01
lyrics_compare_var	0.03	0.03	-0.04
lyrics_conj_avg	0.13	0.03	-0.11
lyrics_conj_var	-0.07	0.12	-0.03
lyrics_death_avg	-0.05	-0.10	0.16
lyrics_death_var	-0.06	-0.07	0.06

lyrics_differ_avg	-0.14	0.05	0.01
lyrics_differ_var	-0.14	0.08	0.02
lyrics_discrep_avg	0.04	-0.02	-0.06
lyrics_discrep_var	-0.05	0.12	-0.03
lyrics_drives_avg	-0.13	0.00	0.04
lyrics_drives_var	-0.13	0.14	0.06
lyrics_family_avg	0.07	0.00	-0.13
lyrics_family_var	-0.03	0.01	-0.07
lyrics_feel_avg	0.07	0.11	-0.13
lyrics_feel_var	-0.05	0.13	-0.16
lyrics_female_avg	0.07	-0.03	-0.16
lyrics_female_var	0.01	0.06	-0.16
lyrics_filler_avg	0.19	-0.08	-0.06
lyrics_filler_var	0.08	-0.06	-0.07
lyrics_focusfuture_avg	-0.02	0.11	0.14
lyrics_focusfuture_var	-0.12	0.18	0.02
lyrics_focuspast_avg	0.19	-0.16	0.05
lyrics_focuspast_var	0.02	0.01	0.09
lyrics_focuspresent_avg	-0.22	0.15	0.14
lyrics_focuspresent_var	-0.07	0.07	0.03
lyrics_friend_avg	0.15	0.01	-0.07
lyrics_friend_var	0.00	0.03	-0.04
lyrics_function._avg	-0.05	0.00	0.07
lyrics_function._var	-0.09	-0.03	0.08
lyrics_health_avg	0.14	-0.06	0.06
lyrics_health_var	0.22	0.05	-0.03
lyrics_hear_avg	0.10	-0.03	0.03
lyrics_hear_var	-0.05	0.23	0.01
lyrics_home_avg	0.13	0.07	-0.06
lyrics_home_var	0.01	-0.03	-0.04
lyrics_i_avg	-0.14	0.01	-0.01
lyrics_i_var	-0.14	0.10	-0.06
lyrics_informal_avg	-0.02	0.12	-0.15
lyrics_informal_var	-0.23	0.16	-0.11
lyrics_ingest_avg	-0.04	-0.01	0.04
lyrics_ingest_var	-0.2	0.09	-0.01
lyrics_insight_avg	0.06	0.01	0.04
lyrics_insight_var	0.00	0.11	0.00
lyrics_interrog_avg	-0.02	0.06	0.07
lyrics_interrog_var	-0.11	0.13	0.05
lyrics_ipron_avg	-0.07	0.08	-0.01
lyrics_ipron_var	-0.06	0.13	-0.11
lyrics_leisure_avg	0.14	-0.04	-0.14
lyrics_leisure_var	0.05	0.01	-0.06

lyrics_male_avg	0.11	-0.05	-0.18
lyrics_male_var	0.16	-0.13	-0.09
lyrics_matches_perc	-0.02	-0.02	-0.01
lyrics_money_avg	0.00	-0.03	0.05
lyrics_money_var	-0.12	0.13	0.04
lyrics_motion_avg	-0.05	0.12	-0.04
lyrics_motion_var	-0.09	0.09	-0.02
lyrics_negate_avg	-0.12	0.02	-0.01
lyrics_negate_var	-0.15	0.26	-0.09
lyrics_negemo_avg	-0.07	-0.17	0.09
lyrics_negemo_var	-0.14	0.01	0.04
lyrics_netspeak_avg	-0.08	0.18	-0.06
lyrics_netspeak_var	-0.22	0.19	-0.09
lyrics_nonflu_avg	0.00	0.16	-0.08
lyrics_nonflu_var	-0.22	0.25	-0.08
lyrics_number_avg	0.13	-0.01	-0.05
lyrics_number_var	-0.11	0.01	0.07
lyrics_percept_avg	0.10	0.06	-0.03
lyrics_percept_var	-0.06	0.07	-0.07
lyrics_posemo_avg	0.20	-0.04	-0.12
lyrics_posemo_var	0.03	0.03	-0.1.
lyrics_power_avg	-0.04	-0.11	0.05
lyrics_power_var	-0.10	0.14	-0.01
lyrics_ppron_avg	-0.12	0.00	-0.06
lyrics_ppron_var	-0.06	0.07	0.01
lyrics_prep_avg	0.03	-0.04	0.15
lyrics_prep_var	-0.01	-0.01	-0.01
lyrics_pronoun_avg	-0.09	0.03	-0.06
lyrics_pronoun_var	-0.09	0.11	-0.02
lyrics_quant_avg	0.08	0.01	-0.11
lyrics_quant_var	0.04	0.08	-0.03
lyrics_relativ_avg	0.09	0.14	0.06
lyrics_relativ_var	0.04	0.00	0.04
lyrics_relig_avg	0.05	-0.01	-0.11
lyrics_relig_var	0.14	0.07	-0.19
lyrics_reward_avg	0.00	0.07	-0.09
lyrics_reward_var	-0.22	0.00	0.06
lyrics_risk_avg	0.03	-0.05	-0.04
lyrics_risk_var	-0.02	0.12	-0.03
lyrics_sad_avg	0.10	-0.04	-0.06
lyrics_sad_var	0.05	0.16	-0.05
lyrics_see_avg	0.15	0.02	0.02
lyrics_see_var	0.04	-0.03	-0.03
lyrics_sexual_avg	0.07	-0.06	-0.13

lyrics_sexual_var	0.12	0.01	-0.23
lyrics_shehe_avg	0.13	-0.01	-0.22
lyrics_shehe_var	0.14	0.05	-0.25
lyrics_social_avg	0.19	-0.04	-0.20
lyrics_social_var	-0.07	0.00	-0.08
lyrics_space_avg	0.08	0.11	0.01
lyrics_space_var	-0.02	0.13	0.05
lyrics_swear_avg	0.05	0.04	-0.15
lyrics_swear_var	-0.13	0.10	-0.07
lyrics_tentat_avg	0.19	-0.02	-0.12
lyrics_tentat_var	0.00	0.07	0.03
lyrics_they_avg	-0.03	-0.14	-0.02
lyrics_they_var	0.08	-0.01	-0.10
lyrics_time_avg	0.07	-0.02	0.13
lyrics_time_var	0.02	-0.02	-0.07
lyrics_verb_avg	-0.07	0.01	0.10
lyrics_verb_var	-0.15	0.04	0.01
lyrics_we_avg	0.00	-0.12	0.15
lyrics_we_var	-0.09	0.06	0.03
lyrics_work_avg	0.11	-0.05	-0.09
lyrics_work_var	0.12	-0.03	-0.15
lyrics_you_avg	-0.01	0.13	-0.08
lyrics_you_var	-0.09	0.09	-0.01
habits_daysMusic_perc	-0.26	0.04	0.13
habits_songs_num	-0.17	-0.05	0.02
habits_songs_DE_perc	0.1	0.02	-0.18
habits_songs_EN_perc	0.00	0.01	0.15
habits_songs_unique_language_num	-0.19	-0.09	0.02
habits_songs_duration_avg	0.05	0.19	-0.08
habits_songs_duration_var	0.11	0.00	-0.08
habits_skipped_songs_num	-0.18	0.01	0.02
habits_skipped_songs_duration_avg	0.02	0.00	-0.02
habits_skipped_songs_duration_var	-0.03	-0.04	0.06
habits_unique_songs_num	-0.06	-0.10	0.00
habits_unique_artists_num	-0.14	-0.05	0.04
habits_unique_albums_num	-0.15	-0.06	0.04
habits_musicapp_totalPerDay_num_avg	-0.17	-0.11	-0.04
habits_musicapp_totalPerDay_num_var	-0.16	-0.09	-0.04
habits_musicapp_totalPerDay_duration_avg	0.08	-0.09	-0.20
habits_musicapp_totalPerDay_duration_var	0.09	-0.13	-0.15
habits_unique_musicapps_num	0.26	-0.17	-0.16
habits_musicapp_session_duration_avg	0.24	-0.03	-0.17
habits_musicapp_session_duration_var	0.21	-0.08	-0.19

Note. Gender was coded with 1 = male and 2 = female. Education was coded as follows: 1 = no school-leaving certificate; 2 = lower secondary school certificate ("Hauptschulabschluss"); 3 = intermediate secondary school certificate ("Realschulabschluss"); 4 = highest secondary school certificate ("Abitur"); 5 = university degree; 6 = doctorate.

Table S3

Permutation-Based Feature Importances for the Random Forest Models

Feature	Rank	$Importance_{mdn}$
audio_liveness_avg	1	-0.016575876
lyrics_social_avg	2	-0.015513118
lyrics_focuspresent_avg	3	-0.013240295
habits_songs_DE_perc	4	-0.007978111
lyrics_social_var	5	-0.007002755
lyrics_Authentic_avg	6	-0.006290838
lyrics_tentat_avg	7	-0.006243536
lyrics_Tone_avg	8	-0.00603504
lyrics_home_avg	9	-0.005886757
habits_songs_duration_avg	10	-0.004704604
lyrics_quant_avg	11	-0.004527771
lyrics_posemo_avg	12	-0.004348704
audio_liveness_var	13	-0.00387039
audio_energy_avg	14	-0.003847714
habits_musicapp_session_duration_var	15	-0.003751723
habits_musicapp_totalPerDay_num_avg	16	-0.003272373
lyrics_shehe_avg	17	-0.003122683
habits_unique_musicapps_num	18	-0.002889304
lyrics_adj_avg	19	-0.002860455
lyrics_verb_avg	20	-0.002856092
lyrics_focusfuture_avg	21	-0.002620412
habits_songs_num	22	-0.002589979
lyrics_informal_avg	23	-0.002587692
lyrics_ipron_var	24	-0.002559751
audio_key_9	25	-0.00255788
lyrics_leisure_var	26	-0.002471277
lyrics_leisure_avg	27	-0.002441874
lyrics_auxverb_var	28	-0.002352145
lyrics_family_var	29	-0.00234942
lyrics_affiliation_avg	30	-0.002290307
lyrics_family_avg	31	-0.001845939
lyrics_Authentic_var	32	-0.001844192
audio_key_3	33	-0.001816007
lyrics_article_avg	34	-0.001757788
lyrics_they_var	35	-0.001756053
lyrics_drives_avg	36	-0.001546544
lyrics_motion_avg	37	-0.001458636
lyrics_power_avg	38	-0.001428681
lyrics_risk_var	39	-0.001341147
lyrics_cogproc_avg	40	-0.001190754
lyrics_i_var	41	-0.001132929

audio_speechiness_avg	42	-0.000982311
lyrics_body_avg	43	-0.000923399
audio_key_5	44	-0.000892439
lyrics_bio_var	45	-0.000862838
lyrics_Sixltr_avg	46	-0.000833984
lyrics_auxverb_avg	47	-0.00083309
lyrics_cogproc_var	48	-0.000803008
habits_daysMusic_perc	49	-0.000774813
lyrics_adverb_var	50	-0.000714314
lyrics_motion_var	51	-0.000655259
lyrics_discrep_avg	52	-0.000565904
lyrics_negemo_avg	53	-0.000535815
lyrics_see_avg	54	-0.000506797
habits_musicapp_totalPerDay_duration_avg	55	-0.000506099
lyrics_anger_avg	56	-0.0005059
lyrics_relativ_avg	57	-0.000446925
lyrics_reward_var	58	-0.000387222
lyrics_adj_var	59	-0.000238381
audio_loudness_var	60	-0.000238169
habits_skipped_songs_duration_avg	61	-0.000208928
audio_instrumentalness_var	62	-0.000208427
lyrics_female_avg	63	-0.000208381
lyrics_certain_var	64	-0.000178518
lyrics_ipron_avg	65	-0.000148731
audio_key_7	66	-0.000148703
lyrics_shehe_var	67	-5.97e-05
habits_musicapp_session_duration_avg	68	-2.91e-05
lyrics_anger_var	69	0
lyrics_anx_avg	70	0
lyrics_ingest_avg	71	0
lyrics_male_avg	72	0
lyrics_money_avg	73	0
lyrics_money_var	74	0
lyrics_nonflu_var	75	0
lyrics_sexual_avg	76	0
lyrics_health_var	77	6.07e-08
audio_mode_1	78	2.96e-05
audio_speechiness_var	79	2.97e-05
habits_songs_unique_language_num	80	5.94e-05
lyrics_article_var	81	5.97e-05
lyrics_relig_avg	82	8.926e-05
lyrics_see_var	83	0.000119091
lyrics_work_avg	84	0.000119204
lyrics_focuspast_var	85	0.000148678

lyrics_power_var	86	0.000148816
lyrics_negate_var	87	0.000148918
lyrics_health_avg	88	0.00020832
lyrics_WC_var	89	0.000238264
audio_instrumentalness_avg	90	0.000297437
lyrics_sad_avg	91	0.000297952
lyrics_WPS_var	92	0.000327207
lyrics_cause_avg	93	0.00032736
audio_matches_perc	94	0.000356837
lyrics_cause_var	95	0.000386522
lyrics_WC_avg	96	0.000416717
lyrics_WPS_avg	97	0.000417146
audio_valence_avg	98	0.000446722
audio_key_0	99	0.000505394
lyrics_compare_avg	100	0.000505918
audio_danceability_var	101	0.000506156
lyrics_interrog_avg	102	0.000506359
habits_musicapp_totalPerDay_num_var	103	0.000535431
audio_key_6	104	0.000535928
lyrics_Tone_var	105	0.000626049
lyrics_percept_var	106	0.000653855
lyrics_netspeak_avg	107	0.000654491
lyrics_feel_var	108	0.000654562
lyrics_informal_var	109	0.00065492
lyrics_pronoun_avg	110	0.000686451
lyrics_assent_var	111	0.000714657
lyrics_work_var	112	0.000714729
audio_tempo_avg	113	0.000744275
lyrics_negemo_var	114	0.000802911
lyrics_relig_var	115	0.000832844
lyrics_we_avg	116	0.000832973
lyrics_compare_var	117	0.000892624
audio_key_4	118	0.000921447
lyrics_achieve_avg	119	0.000922304
audio_loudness_avg	120	0.000922377
lyrics_number_var	121	0.000924448
lyrics_certain_avg	122	0.000952954
audio_key_2	123	0.000953545
lyrics_conj_var	124	0.001010976
lyrics_focuspast_avg	125	0.001012958
habits_songs_EN_perc	126	0.001041577
lyrics_affect_avg	127	0.001042142
habits_skipped_songs_num	128	0.001042195
lyrics_feel_avg	129	0.001071658

lyrics_nonflu_avg	130	0.001100105
lyrics_function._var	131	0.001100307
lyrics_ppron_var	132	0.001101726
lyrics_netspeak_var	133	0.001131042
audio_key_10	134	0.001132291
lyrics_drives_var	135	0.001132728
lyrics_posemo_var	136	0.001159906
lyrics_Clout_var	137	0.00116017
lyrics_number_avg	138	0.001161222
lyrics_prep_var	139	0.001162043
lyrics_Dic_avg	140	0.001189421
lyrics_interrog_var	141	0.001189711
lyrics_adverb_avg	142	0.001190026
lyrics_verb_var	143	0.001220664
lyrics_time_avg	144	0.001220982
lyrics_quant_var	145	0.00122111
lyrics_assent_avg	146	0.001221956
lyrics_you_var	147	0.001249563
lyrics_body_var	148	0.00125039
lyrics_space_var	149	0.001250661
lyrics_differ_var	150	0.001310523
lyrics_space_avg	151	0.001368218
lyrics_achieve_var	152	0.001369717
lyrics_we_var	153	0.001370414
lyrics_discrep_var	154	0.001398501
lyrics_focuspresent_var	155	0.00142786
audio_energy_var	156	0.001518055
audio_danceability_avg	157	0.001518597
lyrics_Dic_var	158	0.001519877
lyrics_Analytic_var	159	0.001547578
habits_songs_duration_var	160	0.001605886
lyrics_prep_avg	161	0.001637957
lyrics_sad_var	162	0.001727029
lyrics_percept_avg	163	0.001753609
audio_key_11	164	0.001756294
lyrics_negate_avg	165	0.001757329
lyrics_risk_avg	166	0.001784586
lyrics_hear_var	167	0.001813596
lyrics_tentat_var	168	0.001815439
lyrics_affiliation_var	169	0.001848278
audio_tempo_var	170	0.001875028
lyrics_focusfuture_var	171	0.001875191
audio_acousticness_var	172	0.001876225
lyrics_reward_avg	173	0.0019667

habits_skipped_songs_duration_var	174	0.002022378
lyrics_Analytic_avg	175	0.002054815
lyrics_i_avg	176	0.002112809
habits_unique_albums_num	177	0.002114949
lyrics_Sixltr_var	178	0.002231035
lyrics_ppron_avg	179	0.002289502
lyrics_conj_avg	180	0.002380104
lyrics_insight_avg	181	0.002381725
lyrics_relativ_var	182	0.002409705
lyrics_insight_var	183	0.002473415
lyrics_differ_avg	184	0.002561003
lyrics_hear_avg	185	0.002592379
lyrics_time_var	186	0.002736402
lyrics_affect_var	187	0.002740161
audio_key_8	188	0.002742303
audio_valence_var	189	0.002887144
lyrics_Clout_avg	190	0.003066065
audio_acousticness_avg	191	0.003095983
lyrics_pronoun_var	192	0.003124383
audio_key_1	193	0.003541482
lyrics_function._avg	194	0.003574831
lyrics_matches_perc	195	0.003601329
lyrics_bio_avg	196	0.003956364
lyrics_you_avg	197	0.006343384

Note. Importancemdn = the median permutation-based feature importance in terms of ρ-loss across the 100 resampling iterations. Importance scores for each iteration are based on median ρ-loss across 10 random permutations.
